# Geospatial technologies for targeting priority areas on surveillance and response of visceral leishmaniasis in São Paulo state, Brazil: embracing a One Health integrative approach

**DOI:** 10.7189/jogh.15.04200

**Published:** 2025-07-25

**Authors:** Rodrigo S Ferro, Elivelton S Fonseca, Felipe L Semensati, Edilson F Flores, Rogério Giufrida, Roberto M Hiramoto, Osias Rangel, Silvia Silva de Oliveira Altieri, Rosana Leal do Prado, Luiz E Prestes-Carneiro

**Affiliations:** 1 Department of Emergency Medicine, School of Medicine, Oeste Paulista University, Presidente Prudente, São Paulo, Brazil; 2 Department of Pos-Graduation, Environment and Regional Development Program, Oeste Paulista University, Presidente Prudente, São Paulo, Brazil; 3 Department of Statistics, School of Sciences and Technology, São Paulo State University, Presidente Prudente, São Paulo, Brazil; 4 Adolfo Lutz Institute (IAL), Parasitology and Mycology Center, São Paulo, São Paulo, Brazil; 5 Pasteur Institute, Disease Control Coordination – State Department of Health, São Paulo, São Paulo, Brazil; 6 Visceral Leishmaniasis Committee, Disease Control Coordination, State Department of Health, São Paulo, São Paulo, Brazil; 7 Center of Epidemiological Surveillance Prof. Alexandre Vranjac, São Paulo, São Paulo, Brazil

## Abstract

**Background:**

In 2023, Brazil accounted for 93.5% of the reported cases of visceral leishmaniasis (VL) in Latin America. This study, employing a One Health approach aims: i) to analyse the spatial distribution of VL using integrated geospatial methods, ii) the temporal trend of VL to assess the impact of the COVID-19 pandemic on the occurrence of cases, and iii) identify spatial clusters of municipalities with heightened environmental vulnerability to prioritise surveillance and control efforts for VL in São Paulo state, Brazil.

**Methods:**

Archival databases from 1999 to 2022 were analysed. High-risk clusters of human VL (HVL) were identified using the Local Moran Index. Incidence and mortality rates were modelled with the Generalized Additive Model. Data on the distribution of *Lutzomyia longipalpis* vectors were obtained from São Paulo’s Supervision in Control of Endemics, while the spatial distribution of canine visceral leishmaniasis (CVL) was based on survey data from the Adolfo Lutz Institute. Environmental factors, including normalized difference vegetation index (NDVI), land surface temperature (LST), and geomorphology, were derived from Moderate Resolution Imaging Spectroradiometer (MODIS) data and Environmental Information Database (BDiA) platform.

**Results:**

*Lutzomyia longipalpis* was detected in 32.4% of municipalities, CVL in 29.0%, and HVL in 18.0%. The western region, characterised by plateau geomorphology, elevated deforestation, and higher temperatures, accounted for 30.6% of high-risk clusters, underscoring its priority status for control and surveillance. While VL cases remain stable during the COVID-19 pandemic, lethality rates increased.

**Conclusions:**

Addressing VL and reducing lethality rates will pose a significant challenge for public health authorities in São Paulo in the coming years.

Visceral leishmaniasis (VL) is a significant vector-borne disease with substantial public health implications worldwide [1]. In 2023, Brazil accounted for 93.5% of reported VL cases in Latin America [2]. In tropical countries, the occurrence and incidence of VL are strongly influenced by climate, vegetation, and socioeconomic development. São Paulo, the most affluent and urbanised state in Brazil, has witnessed the emergence and establishment of urban transmission of VL since the late 1990s [3]. The primary vector, *Lutzomyia longipalpis*, was first detected in an urban area of Araçatuba in 1997, followed by the emergence of Canine Visceral Leishmaniasis (CVL) in 1998 and Human Visceral Leishmaniasis (HVL) in 1999 [4–6]. Situated in the northwest of the state, the Araçatuba region is characterised by a landscape of semi-deciduous seasonal forests at various stages of ecological succession, interspersed with agricultural zones. The region experiences a subtropical climate, with hot, humid summers and mild, dry winters, a pattern shaped by its geographic position within the subtropical zone. Additionally, its extensive road network connecting numerous small towns may facilitate the geographic spread of VL [7–11].

The western region of São Paulo is currently considered an endemic area for VL, with the first autochthonous cases reported in the municipalities of Dracena and Ouro Verde in 2005 [6]. Given that VL is a multifactorial disease [11,12], a combination of socioeconomic and environmental factors such as deforestation, temperature variations, and geomorphological features are believed to contribute to its dissemination throughout this region and beyond.

Since the initial cases in the 1990s, the disease has progressively expanded from the border with the state of Mato Grosso do Sul toward the São Paulo metropolitan region and the coastal zone of the Atlantic Forest [9,10]. However, the ecological heterogeneity across the state highlights several assumptions that remain inadequately explored. The dispersion patterns of VL on a statewide scale are not well understood. Geographic priority areas for surveillance and control measures have yet to be thoroughly identified using high-risk clustering methodologies. Furthermore, environmental factors such as deforestation, temperature, and geomorphology likely influence the distribution and transmission of VL but are poorly characterised. The COVID-19 pandemic may have also influenced the transmission dynamics of VL in São Paulo, as prevention and control activities targeting the vector and urban reservoirs were disrupted during this period. We hypothesised that VL is expanding from the western region, bordering Mato Grosso do Sul state, toward the São Paulo metropolitan area and the Atlantic Forest coastal zone. This spread is likely driven by environmental risk factors such as deforestation, elevated temperatures, and geomorphological features.

Quantitative assessments are essential as analysis and diagnostic instruments advancing the One Health agenda. Geospatial technologies have proven to be critical tools in public health, particularly for vector-borne diseases, by enabling integrated analyses of spatial and epidemiological data [9–11]. These technologies facilitate the storage, analysis, visualisation, and interpretation of information within a georeferenced framework, allowing for a more accurate identification of risk factors and epidemiological patterns [11,12].

Data derived from geomorphology, the study of landforms influenced by climate and hydrology are also crucial for understanding how environmental conditions shape vector distribution and ecology. Previous studies have demonstrated that diseases such as yellow fever, malaria, and leishmaniasis are closely linked to environmental factors, including topographic relief and climate [13–15].

Employing a One Health approach, this study aimed to:

i) to analyse the spatial distribution of VL using integrated geospatial methods;

ii) to analyse the temporal trend of VL to assess the impact of the COVID-19 pandemic on the occurrence of cases of the disease, and

iii) to identify spatial clusters of municipalities with heightened environmental vulnerability to prioritise surveillance and control efforts for VL in São Paulo state, Brazil.

## METHODS

### Study area

As of December 2023, São Paulo state, the richest and most populous state in Brazil, had an estimated population of 47 333 288, representing 21.9% of the country’s total population of 216 284 269, according to the Brazilian Census [16]. São Paulo consists of 645 municipalities and is geographically divided into 15 mesoregions and 18 Regional Networks for Health Assistance (RNHAs).

The western region of São Paulo, classified as endemic for VL, comprises 45 municipalities with an estimated population of 744 219 in 2022, accounting for 16.1% of the state’s total population [17]. This region is administered by RNHA11, headquartered in the municipality of Presidente Prudente. It is characterised by significant socioeconomic challenges, with 86.7% of municipalities having populations below 30 000 residents [17].

Recognising VL as a multifactorial disease that necessitates a One Health approach for a comprehensive understanding, this study analyses several interrelated components involved in its occurrence. Specifically, it examines: the geographic spread of the *Lutzomyia longipalpis* vector; domestic dogs as urban reservoirs; humans as hosts; and environmental variables that influence vector suitability, such as deforestation, land surface temperature, and geomorphology.

Additionally, geospatial methods were applied to analyse statewide drivers of VL clustering, with a particular focus on the western region of São Paulo.

### Data collection

#### Human data

Data on confirmed HVL cases were obtained from the São Paulo State Department of Health, which relies on Brazil’s official reporting system, the Notifiable Diseases Information System (SINAN). The study period spans from 1999 to 2022. It is important to note that SINAN operated in two versions during this time: Sinan-Windows (1998–2006) and Sinan-NET (2007–present). Between 1998 and 2004, confirmed case definitions followed the 1998 Ministry of Health guidelines, relying solely on laboratory criteria (parasitological and/or serological) [18]. From 2005 onwards, a more sensitive case definition was adopted, incorporating clinical-epidemiological criteria. The cumulative spatial distribution of HVL cases was assessed using the municipality-based incidence coefficient, defined as the number of new autochthonous cases per 100 000 inhabitants, calculated based on annual population estimates and municipal land area. The Cumulative Index (CI), a key indicator used in the ‘Visceral Leishmaniasis Action Plan of the State of São Paulo’ (in effect since 2018), was employed in this study as a critical parameter for evaluating disease distribution and control strategies.

Bivariate Local Indicators of Spatial Association (BiLISA) is used to evaluate localised spatial dependence by calculating spatial correlations between one variable (*e.g*. HVL incidence) and the spatially weighted distribution of another variable (*e.g*. CVL incidence). This approach facilitates the identification of high-risk clusters and spatial outliers, offering insights into areas of potential overlap or transmission dynamics. In the univariate spatial analyses, municipalities classified as high-high indicate those with high CVL indices surrounded by neighbours with similarly high indices; the same interpretation applies to the low-low classification. In the bivariate maps, municipalities identified as high-high are those that exhibited high indices for both CVL and HVL, as well as neighboring municipalities with similarly elevated values. Conversely, the low-low classification reflects areas with simultaneously low indices for both indicators and their neighboring municipalities

A Generalized Additive Model (GAM) [19] was employed within a univariate framework to analyse time series data on HVL incidence and mortality rates. Restricted Maximum Likelihood (ReML) was used for smoothing parameter estimation. The mathematical representation of the GAM model is as follows:



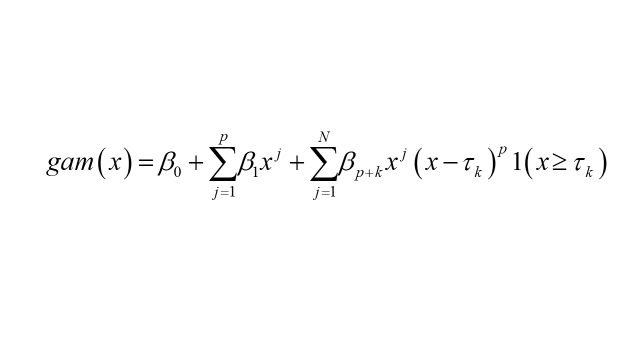



where:

• β0, β1… βn are coefficients of the polynomial part;

• τ are the knots or specific time points where the spline is anchored;

• *P* = polynomial component, and

• x = the predict variable.

The number of basis functions (k) was initially set to 10 but optimised to minimise residual deviance and maximise explained deviance (R^2^). Graphical analysis of curves for k values ranging from 4 to 8 revealed that k = 6 was sufficient to capture the underlying trend without overfitting.

The GAM model was constructed using the Mixed GAM Computation Vehicle (MGCV) package in R [19]. Model adequacy was evaluated through residual analysis, autocorrelation function (ACF) plots at various lags, and the Ljung-Box test to ensure the absence of residual autocorrelation and validate the model's predictive accuracy.

An uninterrupted time series analysis was conducted to assess the temporal trends in HVL cases across the state of São Paulo during the pre-pandemic, pandemic, and post-pandemic periods of the COVID-19 crisis. In this analysis, the number of HVL cases was treated as the dependent variable, with the months of the year serving as the independent variable. The analysis was contextualised within the timeline of the Public Health Emergency of International Concern (PHEIC) for COVID-19, declared by the World Health Organization (WHO) on 11 March 2020, and concluded on 5 May 2023. Accordingly, the pandemic period was defined as spanning from March 2020 to April 2023, totaling 38 months.

Hospital admissions for HVL during the same period were also analysed. While new cases were categorised based on the date of symptom onset, this metric may not accurately reflect the actual time of detection by the health care system due to the chronic progression of the disease. Hospital admission data were obtained from the online repository of the Department of Information Technology of the Unified Health System (DATASUS).

The pre-pandemic period encompassed the 38 months preceding the pandemic, from December 2016 to February 2020. The post-pandemic period was defined as May 2023 to April 2024, based on data availability.

The uninterrupted time series approach enabled the identification of any significant shifts in the temporal trends of HVL incidence across these distinct periods. The number of hospital admissions due to HVL in the same time interval was also assessed, since new cases of the disease were analysed considering the date of onset of symptoms, which may not reflect the actual time when the case was detected by health services, given the chronic nature of the disease. The data source for hospital admissions was extracted from the electronic address of both cases and hospitalisations.

#### Environmental data

Three environmental predictors – geomorphology, land surface temperature (LST), and normalized difference vegetation index (NDVI), were selected for their potential association with the presence of *Lutzomyia longipalpis* and the spread of VL in São Paulo state.

Geomorphology, the study of landforms and their evolution, is a critical factor for understanding how environmental conditions influence VL transmission. São Paulo state is subdivided into five geomorphological compartments: Coastal Plain, Atlantic Plateau, Peripheral Depression, Basaltic Cuestas, and Western Plateau [20,21]. Data on geomorphology were obtained from the Environmental Information and Database (BDiA). These data were imported into QGIS and integrated with maps of municipalities reporting HVL cases. Geomorphological units were highlighted using QGIS symbology tools. Symbology was applied to the geomorphology layer via properties > symbology > style > load style, and the legend was customised to depict the predominant geomorphological units associated with municipalities reporting VL cases.

Land surface temperature (LST) analysis involved the creation of two average temperature maps for daytime using QGIS 3.34. Data were sourced from the United States Geological Survey (USGS) data portal, specifically from TERRA satellite imagery provided by the MODIS instrument.

Land Surface Temperature data were derived using the LST algorithm, which incorporates inputs from bands 31 and 32 (thermal infrared) at a spatial resolution of 1.1 km under clear-sky conditions. Emissivity values were reconstructed using MODIS Level 2 and 3 data, allowing the algorithm to reprocess pixels with known emissivity values. The digital levels (NDs) of bands 31 and 32 represent the energy emitted from the Earth's surface, enabling accurate estimates of surface temperature.

Normalized Difference Vegetation Index (NDVI) was calculated to monitor vegetation cover and assess its role in *Lutzomyia longipalpis* habitat suitability. Orbital images from the MODIS13 sensor (spatial resolution: 1 km) were obtained from 2002 to 2022 and processed using the Cloud-Validated Maximum Value Composite (CVMVC) technique, which minimises cloud interference and ensures high-quality pixel selection.

NDVI was calculated using the following formula:



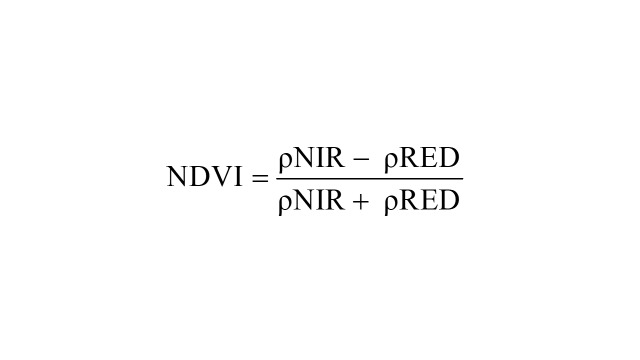



Where ρNIR is the reflectance in the near-infrared band and ρRED is the reflectance in the red band.

NDVI values range from −1 to 1:

• Values close to 1 indicate dense, healthy vegetation.

• Values near 0 indicate areas with little to no vegetation or exposed soil.

• Negative values typically indicate water bodies or clouds.

The NDVI maps, together with LST data, provided valuable insights into the environmental conditions influencing VL transmission and the habitat suitability of *Lutzomyia longipalpis* across São Paulo state.

### Entomological data

Data on the collection and distribution of *Lutzomyia longipalpis* vectors in São Paulo state from 1970 to 2022 were obtained from the Supervision in Control of Endemics (SUCEN) [22]. Vector detection data were compiled and consolidated in the FlebWebLV database. The detection of *Lutzomyia longipalpis* was conducted through systematic entomological surveillance in municipalities across São Paulo. Surveillance involved the use of CDC light mini-traps, which were installed at a density of one trap per household. These traps were placed in environments favorable to vector proliferation, such as areas with decomposing organic material (*e.g*. animal feces, leaves, fruits, and branches) in unpaved backyards, as well as areas with domestic animals like chickens and dogs. Traps were installed at 6:00 pm and exposed for 12 hours.

Specimens were identified using the morphological classification system proposed by Galati [23]. The geographic expansion of *Lutzomyia longipalpis* was analysed based on the year of its first detection in each municipality. Spatial distribution maps were created using QGIS version 10.8 (ESRI 2014, Redlands, California, USA).

### Canine data

Data on the distribution of CVL in São Paulo state from 1998 to 2022 were obtained from the Adolfo Lutz Institute, São Paulo, Brazil. Surveillance of CVL transmission was initiated based on clinical suspicion and conducted by the epidemiological/zoonosis surveillance network in each municipality. Confirmation of CVL cases adhered to Ministry of Health (MoH) guidelines and was achieved through serological testing. Positive results were interpreted based on agreement between the dual-platform rapid immunochromatographic test and enzyme immunoassay. Additionally, parasitological examinations were performed to confirm the presence of the parasite. These diagnostic measures complied with MoH protocols for classifying municipalities as primary or secondary transmission sites.

## RESULTS

### Human data

#### Spatial cumulative distribution, high-risk clusters for HVL transmission in municipalities of São Paulo state, Brazil 1999–2022

Between 1999 and 2022, a total of 3243 autochthonous cases of HVL were reported in São Paulo state. The disease exhibited an eastward expansion, spreading from the western region toward the metropolitan area of São Paulo city. The western region, represented by RHAN11, accounted for 18.7% (606 / 3243) of the reported cases.

**Figure 1:**
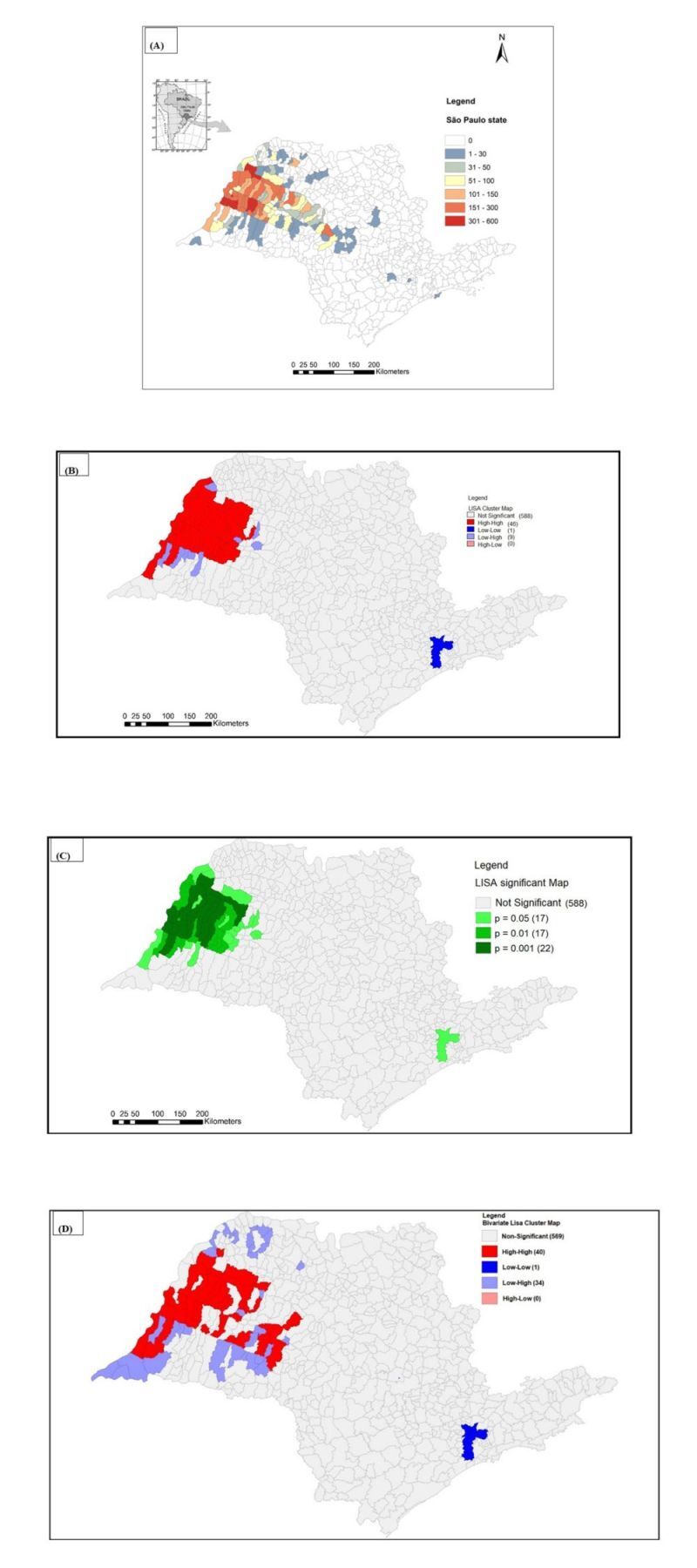
Spatial cumulative incidence and high-risk clusters for Human Visceral Leishmaniasis (HVL) transmission in São Paulo state, 1999–2022. **Panel A.** Cumulative incidence. The colours represent the cumulative number of cases. Most of the municipalities are located in the border of Mato Grosso do Sul spreading from the west to eastern region following the Marechal Candido Rondon Highway. **Panel B.** Spatial distribution of high-risk clusters Local Moran Index with 46/644 classified as high-high munic­ipalities (Local Indicators of Spatial Association – LISA cluster map). **Panel C.** LISA significance map showing that *P* = 0.001 was very significant in 22 municipalities. Both LISA cluster and LISA significance map showed that significant munici­palities are concentrated in the western region of São Paulo state. **Panel D.** In the Bivariate Local Indicator of Spatial Association (BiLISA) Cluster Map in HVL/Canine Visceral Leishmaniasis (CVL) association, 40/644 municipalities were classified as high/high and most of them were located in the western region of São Paulo state.

The mean incidence of HVL was highest in municipalities situated from the northwest to the southwest, along the border of Mato Grosso do Sul (MS) state and in the central region of São Paulo state. Clusters of municipalities with higher cumulative incidences (301–600 cases) were found adjacent to municipalities with lower incidence rates (1–30 cases) (**Figure 1**, Panel A). The western region (RHAN11), particularly municipalities bordering Mato Grosso do Sul and those along the Paraná River basin, had a greater concentration of endemic municipalities. These areas are spatially connected to Minas Gerais state in the north and Paraná state in the south.

Using the univariate Local Moran Index (LISA), 7.1% (46 / 644) of municipalities of Sao Paulo state were classified as high-high risk clusters for HVL. Similarly, the bivariate LISA (BiLISA) analysis identified 6.2% (40 / 644) of municipalities of Sao Paulo state as high-high risk clus­ters (**Figure 1**, Panels B–D).

While the overall prevalence of HVL cases decreased in most municipalities, the number of municipalities reporting new cases showed an upward trend. This spatial expansion primarily occurred through contiguity with adjacent foci, particularly in the central region of the state. However, epidemic leaps were also observed, as infected individuals were detected in distant municipalities where the disease had not previously been established. Notably, cases were reported in the municipality of Guarujá, located on the Atlantic coastal border, as well as in municipalities within the metropolitan area of São Paulo city, far from the endemic epicenter.

#### Yearly incidences and mortality prediction of HVL

**Figure 2:**
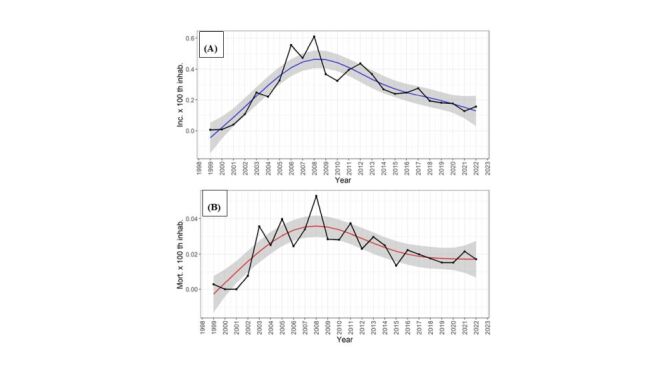
**Panel A.** Actual (black lines) and predicted (blue lines) values for the incidence of HVL in São Paulo state from 1999–2022 by 100 000 inhabitants. **Panel B.** Actual (black lines) and predicted (red lines) values for the mortality of HVL in São Paulo state from 1999–2022 by 100 000 inhabitants. In **Panels A–B** the gray areas applied to the time lines represent the 95% confidence limits for the predicted values.

Temporal trends for HVL incidence and mortality compare actual and predicted values from the models (**Figure 2**, Panels A–B). The coefficients for the temporal models were statistically significant, confirming the reliability of the predictions. The models demonstrated strong goodness-of-fit, with R^2^ values of 0.810 for incidence and 0.642 for mortality. Additionally, the absence of residual autocorrelation further validated the models' accuracy.

Both incidence and mortality curves followed similar trends, with a gradual increase until reaching a peak in 2008. The models predict a decline in both incidence and mortality in the coming years, suggesting progress in controlling the disease.

#### Spatial dependence of HVL cases over 3-year periods

HVL incidence data aggregated into 3-year intervals (**Table 1****)**. The highest number of cases was reported during the periods 2005–2007 and 2009–2010, followed by a gradual decline in subsequent intervals. A notable reduction in cases was observed during 2020–2022 compared to 2017–2019.

**Table 1:** Spatial dependence of human visceral leishmaniasis cases over 3-year periods in São Paulo state, 1999–2022

Three-year period	Cases	Deaths	Lethality (%)	Moran global	Z score	*P-*value
1999–2001	89	8	0.089	0.1328	9.504	0.01
2002–2004	405	49	0.12	0.2987	19.484	0.01
2005–2007	653	48	0.073	0.3791	24.836	0.01
2008–2010	574	52	0.084	0.4598	29.695	0.01
2011–2013	566	45	0.079	0.554	35.59	0.01
2014–2016	378	34	0.085	0.344	22.45	0.01
2017–2019	341	31	0.089	0.270	17.33	0.01
2020–2022	237	26	0.109	0.121	8.36	0.01

The highest Moran Global Index and Z-scores were recorded for the 2011–2013 period, indicating significant spatial clustering during that time. Although a general decline in total case numbers was observed, HVL lethality rates remained consistently high across the study period (2018: 10.2%; 2019: 10.9%; 2020: 10.5%; 2021: 13.8%; 2022: 10.7%), peaking during the pandemic years (2020–2022), which aligns with the period of heightened strain on health services. Throughout the study period, while the overall number of reported cases decreased, the number of municipalities reporting new cases steadily increased. This trend underscores the ongoing geographic expansion of HVL within São Paulo state, highlighting the persistent challenge of controlling its spread.

#### Transmission of human visceral leishmaniasis in COVID-19 pandemic era

**Figure 3:**
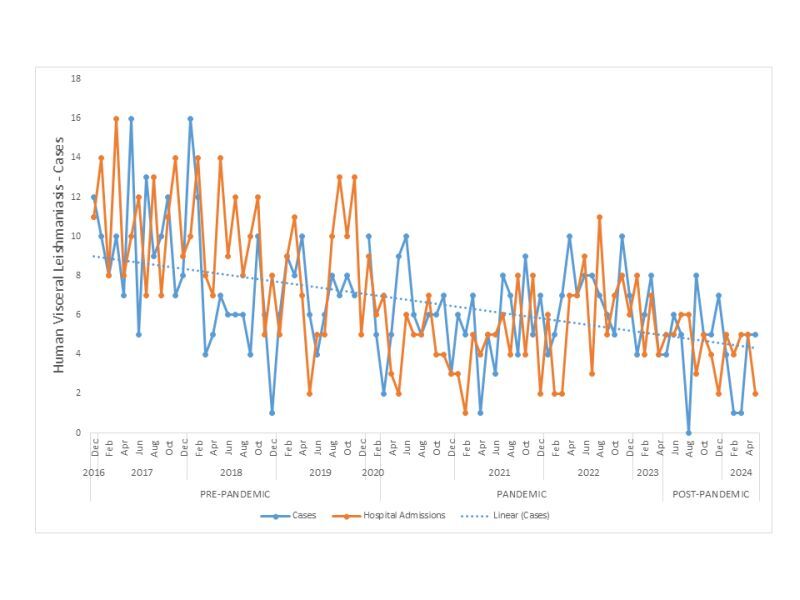
Uninterrupted time series analysis was employed to examine temporal trends in HVL cases across São Paulo state during the pre-pandemic, pandemic, and post-pandemic COVID-19 periods.

Between March 2017 and February 2020, a reduction in the number of notified cases of HVL was observed. During the COVID-19 pandemic era, from March 2020 to March 2022, the number of cases remained stable. However, a significant reduction in notified cases was recorded during the post-pandemic period (**Figure 3**).

This trend underscores the potential impacts of the pandemic and subsequent recovery period on disease surveillance and transmission dynamics. Furthermore, could partially reflect disruptions in data collection due to a shift in priorities to COVID19 pandemic rather than a genuine decline in incidence and reduced health system capacity.

### Environmental data

#### Geomorphology of São Paulo state

**Figure 4:**
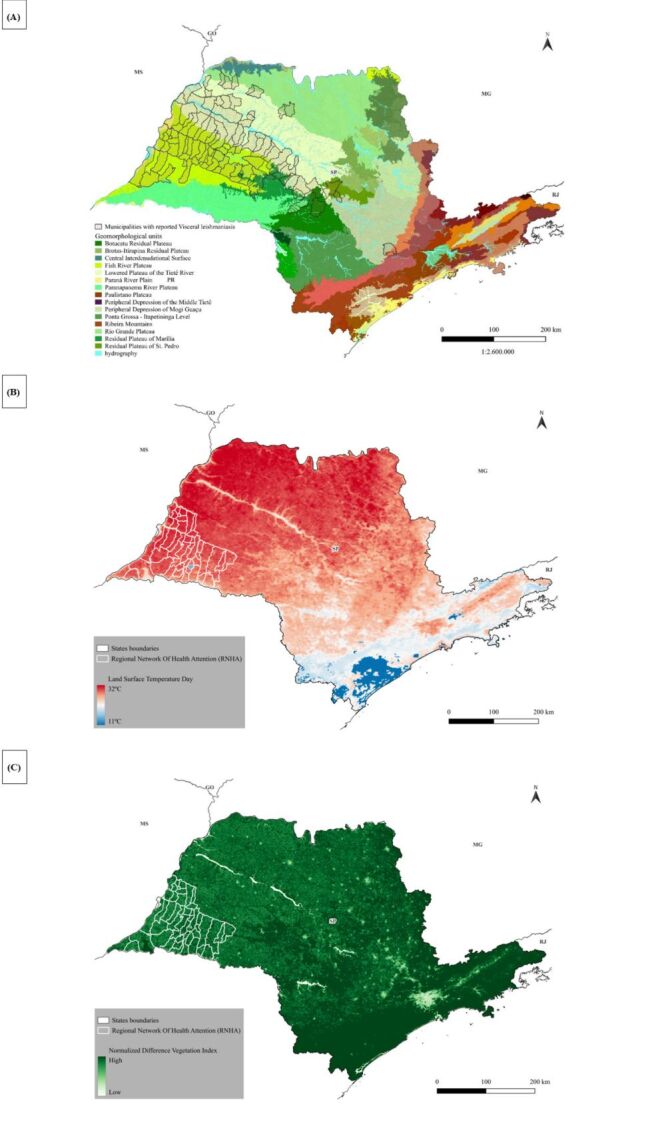
Environmental variables determined in São Paulo state. **Panel A.** The five geomorphological regions of São Paulo state and the municipalities with reported HVL from 1999–2022. **Panel B.** Land surface temperature based on the MODIS sensor for 2000 Source: NASA LP DAAC (2011). MODIS day LST (odds ratio, 4.5) and the 45 municipalities of the RHAN11. **Panel C.** Normalized Difference Vegetation Index in São Paulo state based on the MODIS sensor for 2000–2022, Source: NASA LP DAAC, and the 45 municipalities of the RHAN11. MS – Mato Grosso do Sul state, MG – Minas Geraes state, RJ – Rio de Janeiro state borders.

São Paulo state is divided into five major geomorphological compartments: the Coastal Plain, Atlantic Plateau, Peripheral Depression, Basaltic Cuestas, and Western Plateau (**Figure 4**, Panel A). The Western Plateau, also known as the Chapadas of the Paraná Basin, begins at the border with Mato Grosso do Sul (MS) state and spans the northern, western, and northwestern regions of the state, covering nearly half of its area. Altitudes in this compartment range from 300 to 1000 m and are characterised by undulating relief with flat, wide, and low hilltops. This region is endemic for VL, and *Lutzomyia longipalpis* vectors are prevalent in most municipalities.

The Basaltic Cuestas form the second geomorphological compartment and consist of reliefs with steep slopes on one side and gentle slopes on the other. Volcanic eruptions in this region produced basaltic soils known as Terra Roxa. Altitudes range from 500 to 950 m. The Basaltic Cuestas begin in the plains of the Western Plateau and extend almost continuously from the southern to northeastern parts of the state. Human visceral leishmaniasis has been reported in only a limited number of municipalities within this region, and the spread of *Lutzomyia longipalpis* vectors has been slow, primarily in the central and northeastern areas.

The Peripheral Depression, located between the Basaltic Cuestas and the Atlantic Plateau, is composed predominantly of sediments from the Paraná Basin. This region has altitudes ranging from 500 to 750 m. The spread of *Lutzomyia longipalpis* in the Peripheral Depression is gradual, moving from the southern border with Paraná state toward the northern border with Minas Gerais state. Only a few municipalities in this geomorphological compartment have reported HVL cases.

The Atlantic Plateau, also referred to as the Plateaus and Mountain Ranges of the East-Southeast Atlantic, stretches along the coastline from the south to the northeast. This region is characterised by large mountain ranges composed of crystalline rocks and includes São Paulo’s metropolitan area and the state capital. The terrain has an average altitude of 700 to 800 m, surrounded by the highest peaks of the Atlantic Belt. By 2022, *Lutzomyia longipalpis* was detected in only two municipalities within this compartment, and no HVL cases were reported.

The Coastal Plain forms the fifth geomorphological compartment, extending from the north to the south. It encompasses the coastline, hills, mountain ranges (*e.g*. Serra do Mar, Paranapiacaba, and Itatins), and the Ribeira Valley in the south. Altitudes in this region are below 300 m, with coastal plains formed by sediment deposits carried by rivers and winds from the surrounding mountains. No *Lutzomyia longipalpis* vectors were detected in the Coastal Plain; however, HVL cases were reported in one municipality, likely transmitted by permissive vectors other than *Lutzomyia longipalpis*.

#### Land surface temperature of São Paulo state 2000–2022

Land surface temperature (LST) data revealed distinct patterns across the geomorphological compartments (**Figure 4**, Panel B). The highest temperatures, reaching approximately 32°C, were observed in most areas of the Western Plateau and the northern Basaltic Cuestas. Intermediate temperatures, averaging around 21°C, were recorded in the Peripheral Depression. In the Atlantic Plateau, temperatures averaged 16°C, while the lowest temperatures, approximately 11°C, were found along the Coastal Plain.

An inverse correlation was observed between surface temperature and the presence of *Lutzomyia longipalpis* vectors and HVL cases. Regions with higher temperatures, such as the Western Plateau, were associated with greater vector density and HVL incidence. Conversely, areas with lower temperatures, including the Coastal Plain and parts of the Atlantic Plateau, showed minimal or no vector presence and HVL cases.

#### Normalized difference vegetation index

The vegetation index of São Paulo state, with NDVI values ranging from −1 to 1 (**Figure 4**, Panel C). Higher NDVI values, close to 1, represent dense, healthy vegetation, while values above 0.51 indicate forested areas. Lower NDVI values, below −0.50, correspond to sparse or degraded vegetation.

The Western Plateau exhibited predominantly low NDVI values, reflecting sparse vegetation or degraded biomass. In contrast, increased NDVI values, ranging from 0.7 to 1.0, were recorded in the Basaltic Cuestas and especially in the Peripheral Depression. The Atlantic Plateau and Coastal Plain showed the highest NDVI levels, close to 1.0, indicating dense forest coverage. Interestingly, the São Paulo metropolitan region and the state capital displayed very low NDVI values, close to 0, due to their highly urbanised landscapes, which are surrounded by dense forests in the Atlantic Plateau and Coastal Plain.

An inverse correlation was identified between NDVI levels and the presence of *Lutzomyia longipalpis* vectors and HVL cases. Regions with low NDVI values, such as the Western Plateau, had a higher prevalence of *Lutzomyia longipalpis* and HVL. Conversely, areas with dense vegetation, such as the Atlantic Plateau and Coastal Plain, reported minimal or no vector presence or HVL cases.

Normalized Difference Vegetation Index (NDVI) maps and Land Surface Temperature (LST) data provided critical insights into the environmental conditions associated with VL transmission and the ecological suitability of *Lutzomyia longipalpis* habitats throughout São Paulo state. These environmental variables were temporally aligned with the epidemiological data set through averaging techniques. Although this approach allowed for the identification of broad patterns in vegetation cover and surface temperature, no statistical corrections were applied to address potential temporal mismatches arising from the use of averaged data.

### Entomological data

#### The historical series of *Lutzomyia longipalpis* detection in São Paulo state

By December 2022, the presence of *Lutzomyia longipalpis* was confirmed in 209 out of 645 municipalities (32.4%) (Figure S1, Panel A in the **Online Supplementary Document**). The western region exhibited the largest concentration of municipalities reporting the presence of *Lutzomyia longipalpis*, particularly along the border with Mato Grosso do Sul state and near the Paraná River basin.

The spread of *Lutzomyia longipalpis* from the western region follows a clear geographic pattern along the Marechal Cândido Rondon highway, which traverses São Paulo state from west to east. This highway extends to the metropolitan area of São Paulo, the state capital, appearing to serve as a corridor for the geographic diffusion of vectors toward more urbanised areas in the east.

### Canine data

#### Distribution of canine visceral leishmaniasis in municipalities of São Paulo state, 1997–2022

By December 2022, CVL transmission had been reported in 187 out of 645 municipalities (29.0%) (Figure S1, Panel B in the **Online Supplementary Document**). Similar to the distribution of HVL and *Lutzomyia longipalpis*, the western region exhibited the highest concentration of municipalities with confirmed CVL transmission. The spatial dispersion of CVL mirrors the diffusion pattern of *Lutzomyia longipalpis* vectors following the course of the Marechal Cândido Rondon highway. This pattern suggests that this transportation route plays a critical role in facilitating the spread of CVL toward the metropolitan area of São Paulo. Interestingly, CVL transmission was also observed in municipalities along the coastal area of the Atlantic Ocean, where *Lutzomyia longipalpis* has not been reported. This observation suggests that CVL transmission in these regions may involve infection mediated by permissive vectors other than *Lutzomyia longipalpis*.

## DISCUSSION

By 2022, *Lutzomyia longipalpis* was detected in 32.4% of municipalities, CVL in 29.0%, and HVL in 18.0%. The western region accounted for 18.7% of HVL cases, and BiLISA analysis indicated that this region contains most of the high-risk clusters, characterised by elevated deforestation and temperature. Despite an overall decline in HVL cases over the last decade, particularly during the post-COVID-19 pandemic years (2022–2024), the disease's spatial spread remains a significant public health concern. The rapid expansion of *Lutzomyia longipalpis*, CVL, and HVL across São Paulo state over the past two decades remains only partially understood. Originating from Três Lagoas, an endemic area in Mato Grosso do Sul, the disease has followed an eastward trajectory along municipalities connected by the Cândido Rondon highway-axis (BR300), eventually reaching the metropolitan area of São Paulo [9–11]. Highways appear to facilitate the spread of VL in southeastern and southern Brazil, as highlighted in recent studies [11,24–27]. The expansion of HVL in the state of São Paulo has also been linked to migratory movements, particularly along major transportation corridors. Infrastructure projects such as the construction of the Bolivia-Brazil gas pipeline and the Marechal Rondon Highway have likely contributed to the spread of VL, as migrant workers from Bolivia and Mato Grosso do Sul may have introduced the parasite. This dynamic has been particularly impactful in the western region of the state [9,11]. Spatial analyses indicate that most high-risk municipalities are located within this western region, specifically within the geomorphological Western Plateau, underscoring the critical need to prioritise surveillance and control interventions in this area. The combination of socioeconomic and environmental factors, such as extensive highway networks, low human development indices, and higher temperatures, appears to sustain the disease's transmission cycle [11].

Among environmental risk factors, VL was predominantly concentrated in the Western Plateau, a region characterised by warmer summer temperatures and lower NDVI values, indicative of reduced vegetation cover. Higher temperatures are suggested to influence the life cycle of *Lutzomyia longipalpis* and the development of *Leishmania infantum* within the vector [28]. Similarly, socioeconomic factors, such as the absence of zoonosis control centres and a high prevalence of CVL, contribute to sustained VL transmission [11]. In tropical countries, the occurrence and incidence of VL are strongly influenced by climate, vegetation, and socioeconomic development. This aligns with our findings and those of other studies that have used generalised linear mixed models to quantify the association of socioeconomic and environmental risk factors with VL incidence in São Paulo state [9,11,29,30]. These studies found that VL occurrence was greater in years with higher maximum annual temperatures. Furthermore, a systematic review identified socioeconomic, environmental, and climatic factors as critical determinants of VL incidence in vulnerable populations in tropical and arid developing regions [28].

A particularly notable finding from this study is the limited distribution of *Lutzomyia longipalpis* in municipalities within the Atlantic Plateau and Coastal areas, where HVL cases in humans and dogs were identified. This suggests the involvement of permissive vectors other than *Lutzomyia longipalpis* in disease transmission in these regions. Geographic barriers, such as the Basaltic Cuestas and Peripheral Depression, appear to hinder the expansion of *Lutzomyia longipalpis*. The Basaltic Cuestas, with altitudes between 500 and 950 m, feature mountainous terrain, depressions, and extensive eucalyptus reforestation, which may impede vector dispersal. However, the colonisation and expansion of *Lutzomyia longipalpis* must be understood as occurring across macro, meso, and micro scales, influenced by both broad macroeconomic trends and localised environmental heterogeneity [31]. In the Atlantic Plateau and coastal zones, entomological surveys have identified other sandflies species including *Pintomyia fischeri*, *Migonemyia migonei*, and *Nyssomyia intermedia* that may also play a role in the transmission of CVL and HVL in these areas [9,10,29,30]. Similarly, in the Peripheral Depression, which has altitudes of 500 to 750 m and environmental conditions similar to the Basaltic Cuestas, *Lutzomyia longipalpis* is present in only a few municipalities. In the Peripheral Depression, which ranges in altitude from 500 to 750 m and exhibits environmental conditions similar to those of the Basaltic Cuestas, the presence of *Lutzomyia longipalpis* has been confirmed in only a few municipalities. For instance, in 2008, Cutolo et al. [31] identified a small population of *Lutzomyia longipalpis* sandflies in a cave located within a municipality situated between the Peripheral Depression and Basaltic Cuestas regions. The detection of this vector in areas characterised by sandstone-basalt outcrops suggests a possible ecological association between *Lutzomyia longipalpis* and these geological formations, which may serve as natural reservoirs contributing to the occurrence of VL.

At both regional and statewide scales, VL dispersion predominantly occurs through contiguity. However, evidence also suggests long-distance transmission through epidemic ‘leaps’ as *Lutzomyia longipalpis* has been detected in municipalities far from the endemic epicentre. Canine visceral leishmaniasis often co-occurs in areas where *Lutzomyia longipalpis* is present, and CVL transmission may persist for several years after the vector is first recorded in newly affected municipalities [32]. Regarding CVL diagnosis, the protocols have evolved significantly over time. Since 2008, the Indirect Immunofluorescence Test (IFT) has been employed as both a screening and confirmatory tool to identify non-reactive and infected samples. In 2010, the Enzyme-Linked Immunosorbent Assay (ELISA or EIE) was introduced as the primary screening method, with IFT used for confirmation. A major advance occurred in 2012 with the inclusion of the immunochromatographic rapid test TR-DPP® as a screening tool and EIE as the confirmatory assay. Each protocol iteration has introduced operational improvements and contributed to a reduction in sample rejection rates. The current diagnostic protocol – combining TR-DPP® and EIE – has significantly enhanced the public health response by enabling faster and more accurate CVL diagnoses. This improvement facilitates timely treatment, strengthens disease control strategies, and ultimately supports the broader prevention of VL transmission [33]. Similar to the pattern observed in São Paulo state, the incidence of HVL in Brazil has demonstrated a decreasing trend since 2018, despite intermittent fluctuations and localised outbreaks, particularly in the North and Northeast regions. Although the number of reported cases has declined, HVL remains a significant public health concern, with an average of approximately 2000 cases reported annually [34].

The COVID-19 pandemic did not significantly alter VL transmission dynamics in São Paulo, as notified cases remained stable during the pandemic period. This contrasts with findings from other regions where VL cases declined during 2020 due to disruptions in health care systems [35,36]. 

Globally, the pandemic negatively impacted the management of neglected tropical diseases, leading to the suspension of surveys, active case detection, and drug administration programmes [35,36]. Several risk factors likely contributed to the observed increase in lethality during the pandemic, including delays in diagnosis and treatment, especially among vulnerable groups such as children under five years of age, adults over 50, and individuals with comorbidities or immunosuppressive conditions, such as HIV/AIDS [37]. Between 2021 and 2023 in São Paulo state, 189 individuals were diagnosed with VL; of these, 88 (46.5%) were aged 50 or older, and 26 (13.8%) were coinfected with HIV [38]. These findings are consistent with those of a prior study by Madalosso et al. (2012) [39], which identified 53 deaths (14%) among 376 VL patients between 1999 and 2005 in São Paulo, with lethality disproportionately affecting individuals over 50 years of age.

This study has national and global relevance, particularly for Latin American countries. São Paulo serves as a major crossroads, connecting other Brazilian states such as Mato Grosso do Sul, Paraná, Minas Gerais, and Rio de Janeiro. Additionally, significant population movement occurs between São Paulo and neighbouring countries, including Bolivia, Paraguay, Venezuela, Colombia, and Peru, potentially facilitating the spread of VL beyond São Paulo to other states and nations. The study is innovative in its integration of multiple factors influencing VL transmission, vector dynamics, reservoirs, hosts, and environmental factors [40,41]. To the best of our knowledge, these aspects have not been simultaneously addressed in São Paulo state, offering new insights for the development of effective disease control and prevention strategies. Nevertheless, this study has limitations. Retrospective analyses are inherently susceptible to recall and selection biases and often lack control over confounding variables. Socioeconomic factors, which were not analysed in detail, likely play a critical role in VL transmission. The western region of São Paulo, one of the state’s poorest areas, is particularly vulnerable due to these socioeconomic conditions. The application of the One Health approach relied on archival data, and the concept has not yet been tested in controlled geospatial studies in São Paulo state, partly due to difficulties in obtaining comprehensive databases without financial support. Future research could apply these methodologies using primary data sources to provide a more comprehensive understanding of the factors driving VL transmission.

## CONCLUSIONS

Although the number of HVL cases in São Paulo has declined over time, the continued emergence of cases in new municipalities underscores the disease’s ongoing spatial dissemination and the need for persistent surveillance and targeted public health interventions. Spatial analysis revealed a continuous eastward spread of *Lutzomyia longipalpis*, CVL, and HVL from the western region toward the metropolitan area of São Paulo. This progression is closely linked to environmental factors such as higher temperatures and deforestation, underscoring the significant role of environmental changes in shaping the transmission dynamics of VL. The western region of São Paulo was identified as a critical area, with high-risk clusters requiring priority attention for intensified surveillance and control measures. While the overall number of VL cases has declined, the increasing lethality rates remain a significant concern. Effective control of VL demands a multidisciplinary, One Health approach that integrates human, animal, and environmental health perspectives. Addressing VL transmission challenges will require intersectoral collaboration that extends beyond the traditional health sector. These efforts are vital for improving clinical outcomes and advancing sustainable control strategies for VL in the years ahead.

## Additional material


Online Supplementary Document
