# Supplementary material for: Geospatial technologies for targeting priority areas on surveillance and response of visceral leishmaniasis in São Paulo state, Brazil: embracing a One Health integrative approach
Source: J Glob Health. 2025 Jul 25;15:04200. doi: 10.7189/jogh.15.04200 (PMC12290434; doi:10.7189/jogh.15.04200)

Supplement to: Ferro RS, Fonesca ES, Semensati FL, Flores EF, Giufrida R, Hiramoto RM, Rangel O, de Oliveira Altieri SS, Prestes-Carneiro LE. Geospatial technologies for targeting priority areas on surveillance and response of visceral leishmaniasis in São Paulo state, Brazil: embracing a One Health integrative approach. J Glob Health. 2025;15:04200.

Spatial cumulative distribution, high-risk clusters, yearly incidence, and mortality rates for transmission of human visceral leishmaniasis in municipalities in São Paulo state, Brazil 1999–2022. (A) Cumulative incidence. (B) Spatial distribution of high-risk clusters univariate Moran Index and Local Moran Index High-high municipalities: Ilha Solteira, Itapura, Pereira Barreto, Santo Antonio do Aracangua, Castilho, Andradina, Murutinga do Sul, Guaracai, Mirandopolis, Lavinia, Valparaiso -Rubiacea, Guararapes, Aracatuba, Bilac, Coroados, Bento Abreu, Nova Independencia, Piacatu, Iacri, Rinopolis, Parapua, Salmorao, Osvaldo Cruz, Sagres, Lucelia, Pracinha, Adamantina, Mariapolis, Florida Paulista, Pacaembu, Flora Rica, Irapuru, Junqueiropolis - Monte Castelo, Tupi Paulista, Dracena, São Joao do Pau D'alho -Nova Gautaporanga, Ouro Verde, Santa Mercedes, Pauliceia, Panorama, Presidente Epitacio, and Presidente Venceslau. Low-high: Suzanopoli, Gabriel Monteiro, Caiua, Piquerobi -Ribeirao dos Indios, Emilianopolis, Presidente Prudente, Glicerio, Alto Alegre. Low-low: São Paulo. C) (LISA) clusters; D) LISA Bivariate Cluster Map (BILISA)

High-high: 40 municipalities: Lavinia – Bento de Abreu – Rubiceia – Coroados – Junqueiropolis – Santa Mercedes – Irapuru – Bilac – Tupi Paulista – Pacaembu – Lins – Aparecida D´oeste – Pereira Barretos – Panorama – Ouro Verde – Andradina – Santo Antonio de Arancangua – Araçatuba – Mirandopolis – Guaraçai – Dracena – Valparaiso – Buritama – Pauliceia – Nova Independencia – Salmourão – Monte Castelo – Piacatu -

Presidente Epitácio – Osvaldo Cruz – Presidente Venceslau – Flora Rica – Getulina - Mariapolis – Sagres – Tupã – Pompéia – Marília – Bastos – Marabá Paulista.

Low-high: 34 municipalities: Santa Rita D´oeste – Macedonia – Guarani D´oeste – Caiuá – Urania – Pedranópolis – Rubineia – Piquerobi – Ribeirão dos Índios – Estrela D´oeste – Aspasia – Emilianópolis - Queiroz – Meridiano – São João das duas Pontes – Herculândia – São João de Iracema – Balsamo – Suzanópolis – Júlio Mesquita – Brejo Alegre – Rancharia – Quata – Oriente – Lutécia – Teodoro Sampaio – Gabriel Monteiro – Oscar Bressane – Mirante do Paranapanema – Echaporã – Rosana – Aguas de São Pedro – Euclides da Cunha Paulista – Bora.

Low-low: 1 municipality: São Paulo.

High-low: 0

Figure S1. (A) Distribution of *Lutzomyia longipalpis* in municipalities of São Paulo state from 1970-2022 (B) Distribution of canine visceral leishmaniasis in municipalities of São Paulo state from 1998–2022.

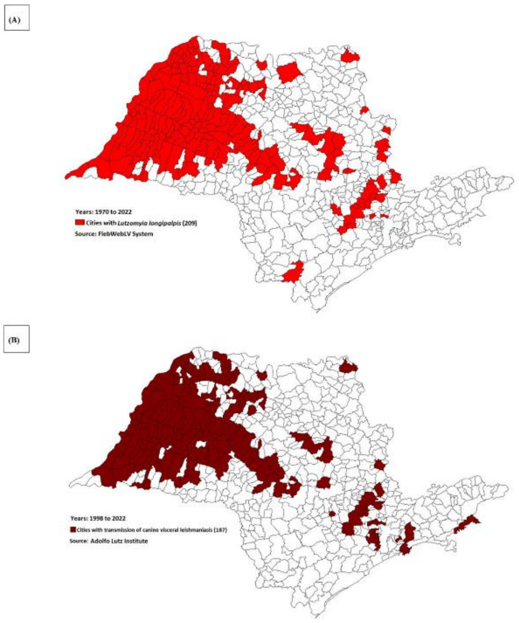

Supplement: Online Supplementary Document [file jogh-15-04200-s001.pdf]
